# Can CT Pelvimetry Be Used to Predict Circumferential Resection Margin Positivity in Laparoscopic Resection of Middle and Lower Rectum Cancer?

**DOI:** 10.7759/cureus.31745

**Published:** 2022-11-21

**Authors:** Afig Gojayev, Cemil Yuksel, Serkan Akbulut, Ogün Erşen, Batuhan Bakırarar, Başak Gülpınar, Ayşegül Gürsoy Çoruh, Ali Ekrem Unal, Salim Demirci

**Affiliations:** 1 Department of General Surgery, Baskent University School of Medicine, Ankara, TUR; 2 Department of Surgical Oncology, Mersin State Hospital, Mersin, TUR; 3 Department of Surgical Oncology, Ankara University School of Medicine, Ankara, TUR; 4 Department of General Surgery, İzmir Ekol Hospital, İzmir, TUR; 5 Department of Biostatistics, Ankara University School of Medicine, Ankara, TUR; 6 Department of Radiology, Ankara University School of Medicine, Ankara, TUR

**Keywords:** tomography, rectal cancer, positive surgical margin, pelvimetry, laparoscopy

## Abstract

Background

Previous studies have shown that pelvimetry can be valuable in predicting surgical difficulties in rectal cancer operations. However, its usability in predicting circumferential resection margin (CRM) involvement remains debatable. This study investigated the factors affecting CRM status and the importance of computed tomography (CT) pelvimetry in predicting CRM involvement in laparoscopic resection of middle and lower rectal cancer.

Methodology

In this study, we retrospectively investigated the data of 111 patients who underwent a laparoscopic operation for middle and lower rectum cancer at Ankara University Faculty of Medicine, Department of Surgical Oncology between January 2014 and January 2020. The predictive value of CT pelvimetry and other variables on the CRM status was analyzed.

Results

The following four pelvic parameters differed significantly between the genders: transverse diameter of the pelvic inlet (p = 0.024), anteroposterior diameter of the pelvic outlet (p = 0.003), transverse diameter of the pelvic outlet (p < 0.001), and pelvic depth (p < 0.001). The effect of pelvic anatomic parameters on CRM involvement was not found to be significant. It was found that tumor height from the anal verge (p = 0.004), tumor size (p < 0.001), and gender (p = 0.033) were significant risk factors for CRM involvement. Survival was poor in patients with male gender (p = 0.032), perineural invasion (p < 0.001), and grade 3 tumor.

Conclusions

In this study, no benefit was found in predicting CRM positivity from CT pelvimetry in the laparoscopic resection of middle and lower rectal cancer. Besides, tumor height from the anal verge, tumor size, and gender were important factors for CRM positivity. Although our study sheds light on this issue, prospective randomized studies with larger sample sizes are needed.

## Introduction

The frequency of rectal cancer is gradually increasing among cancers of the gastrointestinal system. It has been stated in previous studies that the male gender has a negative impact on survival in colorectal cancer [1]. The deep and narrow pelvis in men causes inadequate surgical resection which causes pelvic recurrence to be higher in men. Previous studies described that circumferential resection margin (CRM) positivity is associated with local recurrence [2]. CRM is the closest radial border between the deepest invasion of the tumor and the edge of the soft tissue resected around the rectum and should be measured in millimeters [3]. According to the European Society for Medical Oncology (ESMO) guidelines, CRM positivity is defined when it is less than 1 mm. CRM positivity is associated with an unfavorable prognosis [4]. Surgical margin positivity is related to many factors other than the patient’s pelvic anatomy. Tumor size, mesorectal volume, surgical technique, and surgeon’s experience can be cited as examples of these factors. In recent studies, it has been proven in histological specimens that the CRM, which is at risk for tumor positivity, can be shown on magnetic resonance imaging (MRI) scanning before surgery [5-7].

Although it has been shown in most studies that pelvimetry can be valuable in predicting surgical difficulties such as the duration of surgery and the amount of bleeding in rectal cancer surgery, its predictive value in surgical margin positivity has not been clarified yet.

## Materials and methods

This study was approved by the Ethics Committee of our institution (decision no: I1-41-20). We retrospectively investigated the data of 111 patients who underwent laparoscopic operation for middle and lower rectum cancer at Ankara University Faculty of Medicine, Department of Surgical Oncology between January 2014 and January 2020. The inclusion criteria were as follows: availability of complete computed tomography (CT) data, patients with middle (5.1-10 cm) and lower (0-5 cm) rectal tumors detected on preoperative colonoscopy, and patients with a preoperative diagnosis of pathologically proven rectal adenocarcinoma. Cases with missing file data records, inaccessible CT images, and cases that were converted from laparoscopy to open surgery were excluded. Along with the pelvic anatomical parameters of the patients, parameters such as age, gender, body mass index (BMI), neoadjuvant therapy status, tumor size, the distance of tumor from the anal verge, tumor staging, pathological CRM status, local recurrence, distant metastasis, and operation type were also evaluated. Pelvimetric measurements were made by radiologists working in our institution by examining CT images.

**Figure 1:**
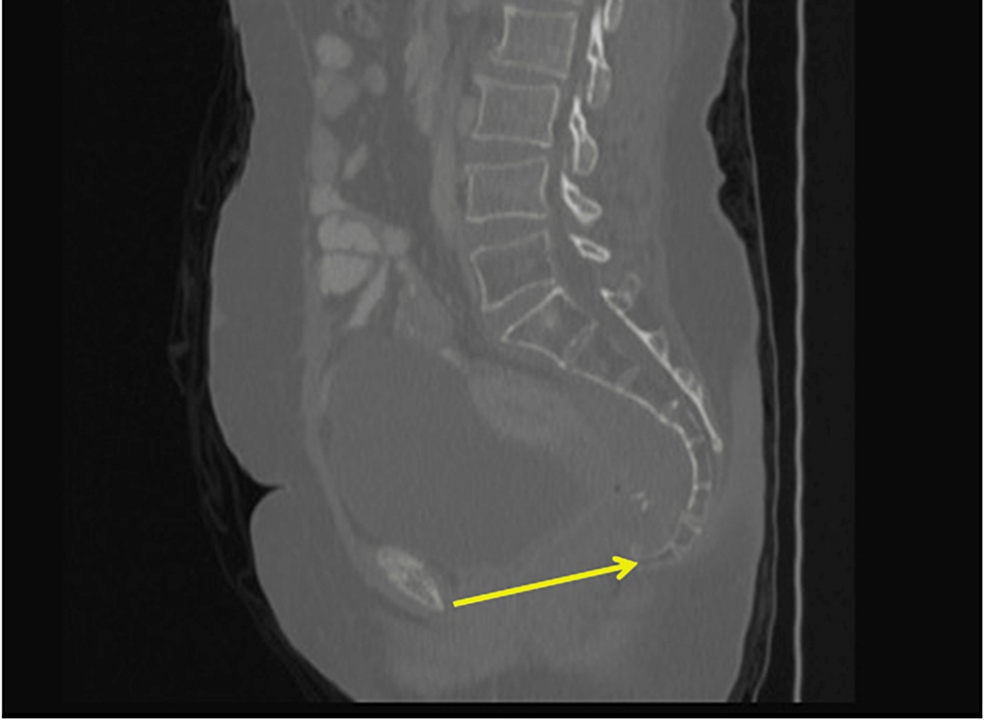
Anteroposterior diameter of the pelvic outlet

The following five pelvic parameters (we used these five pelvic parameters as they represent the diameters of the pelvic inlet and outlet) were measured: 1. Anteroposterior diameter of the pelvic inlet: axis from the superior aspect of the pubic symphysis to the sacral promontory (Figure 1).

**Figure 2:**
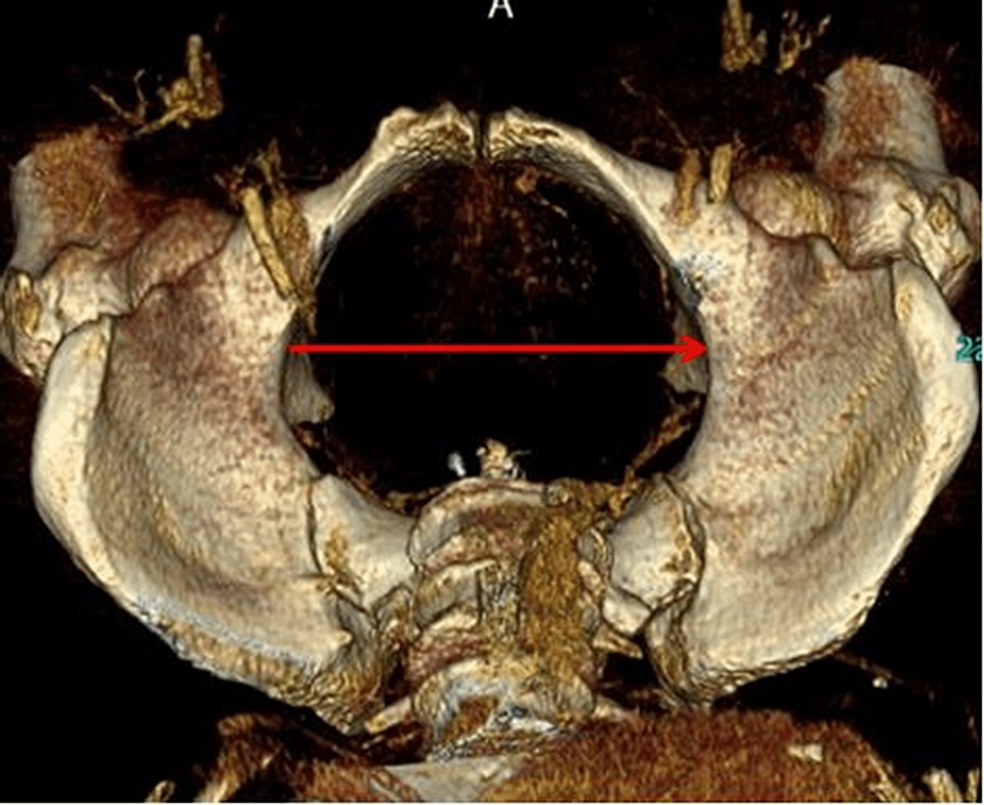
Transverse diameter of the pelvic inlet.

**Figure 3:**
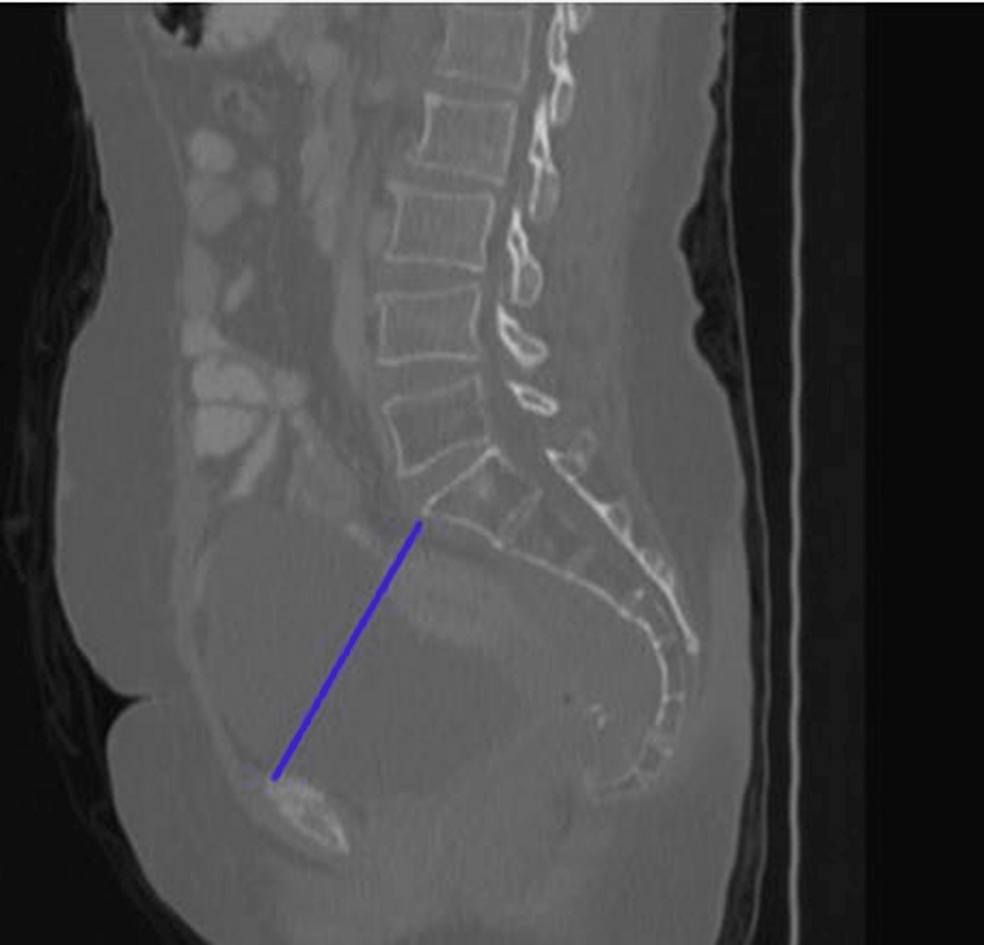
Anteroposterior diameter of the pelvic inlet.

**Figure 4:**
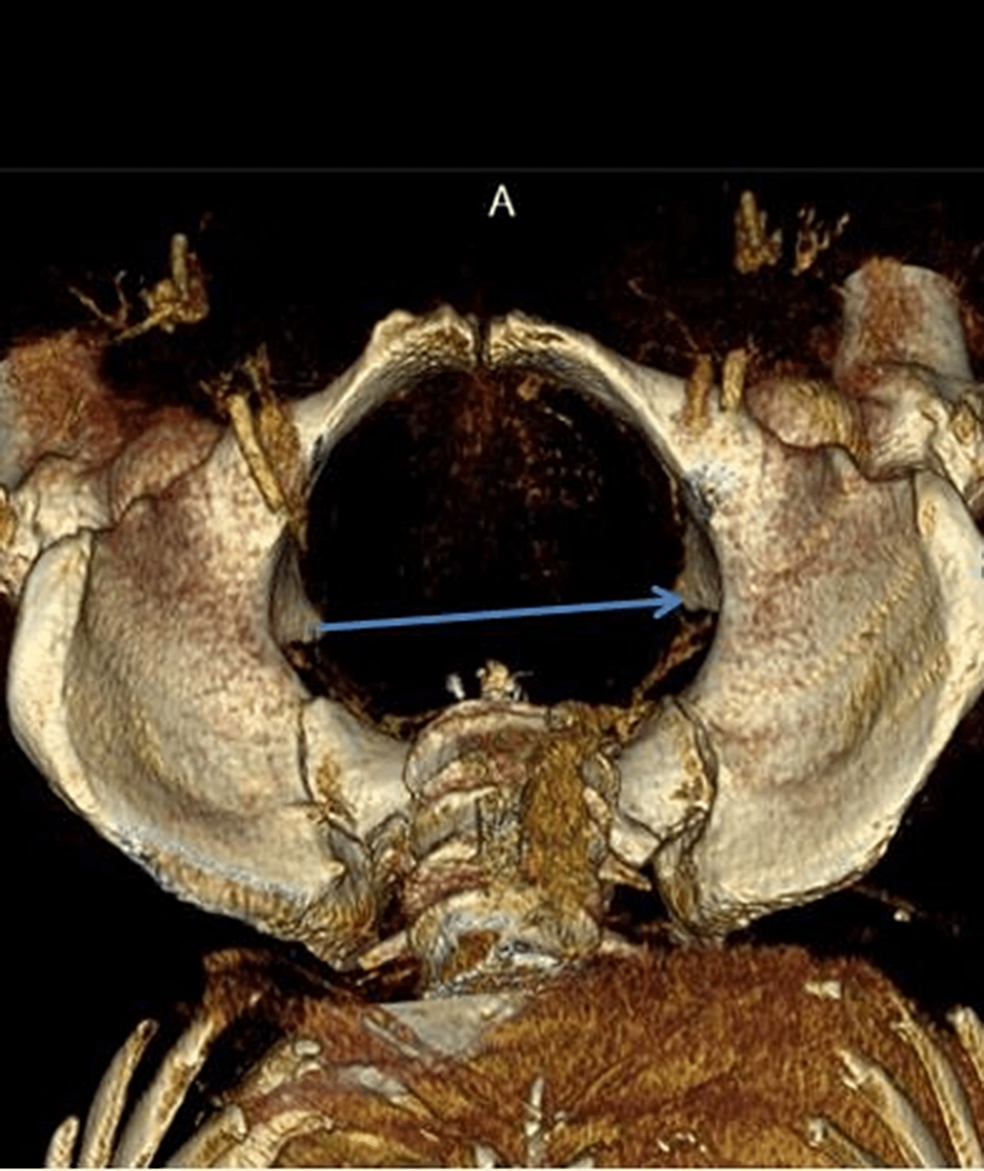
Transverse diameter of the pelvic outlet.

**Figure 5:**
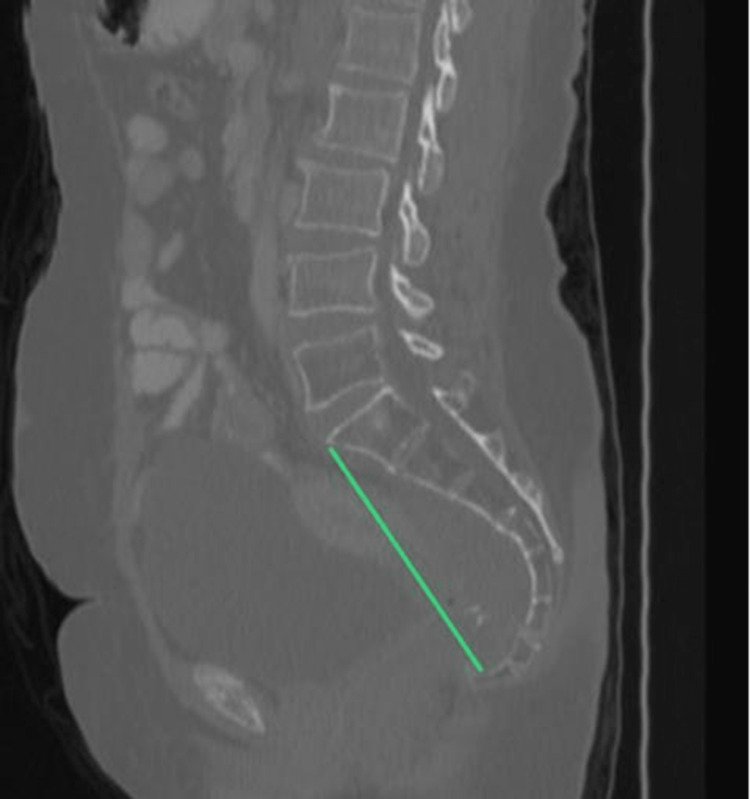
Pelvic depth.

2. Transverse diameter of the pelvic inlet: the longest lateral axis in the iliopectineal line (Figure 2).

3. Anteroposterior diameter of the pelvic outlet: the axis from the inferior aspect of the pubic symphysis to the tip of the coccyx (Figure 3).

4. Transverse diameter of the pelvic outlet: the distance between the tips of the ischial spines (Figure 4).

5. Pelvic depth: distance between the sacral promontory to the tip of the coccyx (Figure 5).

Statistical analysis

SPSS 11.5 software was used for data analysis. The mean ± standard deviation and median (minimum-maximum) were used for the quantitative variables and the number of patients (percentage) for the qualitative variables. The difference between the categories of the qualitative variable with two categories in terms of the quantitative variable was examined using the Mann-Whitney U test because the assumptions of normal distribution were not provided. Univariate and multivariate logistic regression analyses were used to determine the risk factors affecting the CRM status. Kaplan-Meier method was used for survival analysis. Statistical significance was denoted by p < 0.05.

## Results

In total, 53 (47.7%) patients were females, and 58 (52.3%) were males. A total of 55 (49.5%) cases were located in the lower rectum, and 56 (50.5%) were in the middle rectum region. Overall, 84 (75.7%) patients underwent low anterior resection (LAR), and 27 (24.3%) patients underwent abdominoperineal resection (APR). Mean ± SD and median (minimum-maximum) values of tumor size ​​were 4.25 ± 1.70 and 4.00 (1.00-8.00) cm, respectively. When the pathological tumor stage was examined, most cases were stage IIIB (49 cases; 44.1%). Only one patient had stage IIC (0.9%) rectal cancer. CRM was clear in 81.1% of the patients. Examining the long-term results, 11 (9.9%) patients developed local recurrence, and 32 (28.8%) patients had distant metastasis. Table 1 shows the demographic, surgical and pathological characteristics, and long-term results of the patients.

**Table 1:** Patient characteristics. Values are expressed as mean ± standard deviation, or n (%). SD: standard deviation; BMI: body mass index; CRT: chemoradiotherapy; CRM: circumferential resection margin; LAR: low anterior resection; APR: abdominoperineal resection

Variables		
Age (years)	Mean ± SD	58.92 ± 12.05
	Median (minimum-maximum)	57.00 (33.00-88.00)
Gender, n (%)	Male	58 (52.3)
	Female	53 (47.7)
BMI (kg/m^2^)	Mean ± SD	23.86 ± 4.31
	Median (minimum-maximum)	23.00 (17.00-35.00)
Neoadjuvant CRT, n (%)	(-)	61 (55.0)
	(+)	50 (45.0)
Tumor height from the anal verge, n (%)	Lower	55 (49.5)
	Middle	56 (50.5)
Surgical procedure, n (%)	LAR	84 (75.7)
	APR	27 (24.3)
Tumor staging, n (%)	I	15 (13.5)
	IIA	34 (30.7)
	IIB	4 (3.6)
	IIC	1 (0.9)
	IIIA	2 (1.8)
	IIIB	49 (44.1)
	IIIC	6 (5.4)
Tumor size	Mean ± SD	4.25 ± 1.70
	Median (minimum-maximum)	4.00 (1.00-8.00)
Local recurrence, n (%)	(-)	100 (90.09)
	(+)	11 (9.90)
Distant metastasis, n (%)	(-)	79 (71.2)
	(+)	32 (28.8)
CRM status, n (%)	Clear	90 (81.1)
	Involved	21 (18.9)

In this study, a significant difference was found between genders in the following four pelvic parameters: transverse diameter of the pelvic inlet (p = 0.024), anteroposterior diameter of the pelvic outlet (p = 0.003), transverse diameter of the pelvic outlet (p < 0.001), and pelvic depth (p < 0.001). These findings are shown in Table 2.

**Table 2:** The pelvic anatomical parameters of the patients. Values are expressed as mean ± standard deviation, or n (%). SD: standard deviation

Variables	Gender				
	Male		Female		
	Mean ± SD	Median (minimum-maximum)	Mean ± SD	Median (minimum-maximum)	P-value
Anteroposterior diameter of the pelvic inlet	109.83 ± 11.27	107.00 (88.70-138.00)	112.68 ± 8.48	110.80 (97.00-130.50)	0.082
Transverse diameter of the pelvic inlet	130.19 ± 8.55	128.90 (111.60-145.00)	134.22 ± 7.39	133.70 (123.60-154.20)	0.024
Anteroposterior diameter of the pelvic outlet	91.04 ± 12.74	88.00 (69.00-112.60)	97.06 ± 8.48	97.10 (84.00-118.00)	0.003
Transverse diameter of the pelvic outlet	102.01 ± 8.76	102.30 (85.00-117.00)	111.38 ± 11.07	109.50 (84.00-141.40)	<0.001
Pelvic depth	123.58 ± 10.08	123.50 (100.50-145.00)	114.74 ± 12.07	111.40 (92.00-138.40)	<0.001

Considering the results of univariate logistic regression analysis in Table 3, tumor distance from the anal verge (p = 0.003), transverse diameter of the pelvic inlet (p = 0.016), tumor size (p < 0.001), and gender (p = 0.019) were significant risk factors and were included in the multivariate logistic regression analysis.

**Table 3:** Univariate logistic regression results for circumferential resection margin status. β: beta coefficient; SE: standard error of mean; OR: odds ratio; BMI: body mass index; LAR: low anterior resection; APR: abdominoperineal resection; CI: confidence interval

Variables		β	SE	P	OR	95% CI	
						Lower limit	Upper limit
Age (years)		0.024	0.020	0.239	1.024	0.984	1.066
BMI (kg/m^2^)		0.033	0.055	0.546	1.034	0.928	1.152
Tumor height from the anal verge (middle)	Lower	1.761	0.595	0.003	5.816	1.811	18.678
Surgical procedure (LAR)	APR	0.273	0.544	0.615	1.314	0.453	3.814
Anteroposterior diameter of the pelvic inlet		-0.045	0.028	0.108	0.956	0.906	1.010
Transverse diameter of the pelvic inlet		-0.080	0.034	0.066	0.923	0.864	0.985
Anteroposterior diameter of the pelvic outlet		-0.012	0.022	0.579	0.988	0.948	1.031
Transverse diameter of the pelvic outlet		-0.032	0.024	0.187	0.969	0.925	1.015
Pelvic depth		0.029	0.021	0.174	1.029	0.987	1.072
Tumor size (<5 cm)	≥5 cm	2.338	0.661	<0.001	10.364	2.838	37.847
Gender (Female)	Male	1.297	0.554	0.019	3.657	1.234	10.836

As shown in Table 4, tumor height from the anal verge, tumor size, and gender were significant together. The lower location of the rectal tumors increases the risk of CRM status being involved by 6.436 times. Tumor size ≥5 cm increases the risk of CRM status being involved by 11.597 times. Male gender increases the risk of CRM status being involved by 3.841 times.

**Table 4:** Multivariate logistic regression results for circumferential resection margin status. β: beta coefficient; SE: standard error of mean; OR: odds ratio; CI: confidence interval

Variables		β	SE	P	OR	95% CI	
						Lower limit	Upper limit
Constant		-5.003	0.948	<0.001	-	-	-
Tumor height from the anal verge (middle)	Lower	1.862	0.655	0.004	6.436	1.784	23.221
Tumor size (<5 cm)	≥5 cm	2.451	0.703	<0.001	11.597	2.925	45.984
Gender (female)	Male	1.346	0.630	0.033	3.841	1.118	13.194

Kaplan-Meier analysis results are summarized in Table 5. Survival was poor in patients with male gender (p = 0.032), perineural invasion (p < 0.001), and grade 3 tumor (p = 0.016). The effect of age (p = 0.785), tumor height from the anal verge (p = 0.393), tumor size (p = 0.088), lymphovascular invasion (p = 0.190), and CRM status (p = 0.890) on survival was not statistically significant.

**Table 5:** Kaplan-Meier analysis results. SD: standard deviation; CRM: circumferential resection margin

Variables		Survival					
		1 year (%)	3 years (%)	5 years (%)	Lifetime		P-value
					Mean ± SD	Median ± SD	
General		95.5	85.9	74.6	95.07 ± 5.54	136.00 ± 0.01	-
Age (years)	≤65	96.2	82.0	71.6	95.76 ± 6.86	136.00 ± 0.01	0.785
	>65	93.9	93.9	81.3	64.39 ± 3.50	69.00	
Gender	Male	96.6	84.6	65.9	57.55 ± 2.88	69.00 ± 3.11	0.032
	Female	94.3	86.8	83.3	108.12 ± 7.67	136.00 ± 0.01	
Tumor height from the anal verge	Lower	94.5	81.8	75.5	101.07 ± 8.06	136.00 ± 0.01	0.393
	Middle	96.4	89.3	73.9	61.31 ± 2.83	69.00	
Tumor size (cm)	<5	95.0	93.3	80.3	102.42 ± 7.25	136.00 ± 0.01	0.088
	≥5	96.1	77.9	68.5	56.30 ± 3.26	69.00	
Lymphovascular invasion	(-)	96.7	91.7	77.6	62.96 ± 2.51	-	0.190
	(+)	94.1	79.8	72.9	88.58 ± 8.98	69.00 ± 11.75	
Perineural invasion	(-)	97.4	93.4	81.8	66.14 ± 2.18	-	<0.001
	(+)	91.4	69.0	56.5	65.77 ± 10.10	62.00 ± 22.51	
Grade	1	-	90.9	81.8	64.73 ± 4.49	69.00	0.016
	2	94.5	91.8	79.1	63.96 ± 2.60	-	
	3	96.3	62.5	54.7	68.18 ± 12.10	69.00 ± 14.07	
CRM status	Clear	95.6	85.0	72.8	95.33 ± 5.87	136.00 ± 0.01	0.890
	Involved	95.2	90.5	90.5	59.76 ± 4.22	62.00 ± 17.21	

## Discussion

CRM is an important prognostic factor for survival as pathological TNM, as previously described [8]. CRM involvement is an indicator of poor prognosis and may cause a high rate of local recurrence [8,9]. CRM positivity is affected by many factors. Pelvic anatomy, tumor size, mesorectal volume, the distance of other pelvic organs to the rectal tumor, surgical technique, and the surgeon’s experience are some examples of these factors [10]. High positive CRM rates in rectal cancer operations may be related to difficulties in obtaining intact total mesorectal excision (TME). It is technically more difficult to perform TME in low rectal tumors compared to middle rectal tumors. This difficulty may be related to the pelvic anatomy. Some authors have used MRI to evaluate pelvimetry. According to previous studies, the pelvis is narrower and deeper in men compared to women. At the same time, rectal surgery in men is more difficult because the male mesorectal volume is larger [11,12]. The correlation between the difference in pelvic parameters and CRM status remains controversial.

Pelvimetry has been widely used to estimate cephalopelvic disproportion in pregnant women before delivery [13]. As previously described, CT or MR pelvimetry is an effective method for measuring pelvic parameters used in rectal cancer surgery [14-16]. The cost of MRI pelvimetry is higher than CT techniques, which limits the clinical use of MRI. Therefore, CT pelvimetry is used more frequently in rectal cancer patients due to its relatively inexpensive cost and convenience.

Verschueren et al. showed that there are significant differences in pelvic measurements between genders [17]. In our study, four pelvic parameters that showed significant differences between the genders were the transverse diameter of the pelvic inlet (p = 0.024), anteroposterior diameter of the pelvic outlet (p = 0.003), transverse diameter of the pelvic outlet (p < 0.001), and pelvic depth (p < 0.001). This result is in line with previous studies [17-19]. Colorectal surgeons say that when performing rectal cancer surgery, it is generally easier to work in a female pelvis than in a male pelvis [17-19]. Laparoscopic operation for rectal cancer is more difficult in men because the male pelvis is narrow and deep. It is probably for this reason that in the present study, CRM status was affected by gender, and significantly higher CRM involvement was found in men (p = 0.019). Boyle et al. and Baik et al. showed that a narrow pelvis can increase the rate of anastomotic leak and positive CRM, leading to poor surgical outcomes [11,14]. This may be related to the fact that rectal surgery in the deep and narrow pelvis is technically hard and troublesome.

In this study, according to multivariate logistic regression results for CRM status, tumor height from the anal verge, tumor size, and gender were found to be significant. There was no significant relationship between pelvic anatomical parameters and CRM status. This is in line with the study by Salerno et al. [10]. They described that the only predictive factor for CRM involvement was the tumor height from the anal verge, and at the same time, MR pelvimetry and CRM involvement had no significant relationship.

In this study, no significant relationship was found between BMI and CRM positivity. This result is consistent with those reported by Kang et al. [2]. In contrast, Atasoy et al. found a significant relationship between high BMI and CRM involvement [20].

In this study, CRM positivity was more frequent in patients with tumor size ≥5 cm (p < 0.001). There are studies in the literature with similar results [2].

Obtaining clear CRM in rectal cancer operation is important to protect against the development of local recurrence. CRM status can be affected by both the experience of the surgeon and the technical aspects of the surgery. In the study by Eriksen et al., the rate of local recurrence was found to be higher in patients who underwent APR than in those who underwent LAR [21]. This result was attributed to the more frequent tumor perforation during APR [21,22]. Similarly, the Dutch TME Study found higher CRM involvement after APR [23]. However, in the present study, the type of surgery had no effect on CRM status (p = 0.615). This is in line with previous studies [2,20]. Besides, some authors do not recommend a laparoscopic method for rectal malignancy in a narrow pelvis [24-26]. Xu et al. reported that transanal TME is superior and more reliable in obtaining clear CRM than laparoscopic TME [27]. Because of these promising results, transanal TME has recently become an increasingly preferred technique.

In this study, survival was poor in patients with male gender (p = 0.032), perineural invasion (p < 0.001), and grade 3 tumors. However, the effect of age (p = 0.785), tumor height from the anal verge (p = 0.393), tumor size (p = 0.088), lymphovascular invasion (p = 0.190), and CRM status (p = 0.890) on survival was not statistically significant. Similar to the present study, Khani, et al. showed that there was no significant difference in survival between patients with and without CRM involvement [28]. However, studies in the literature suggest that CRM status is a critical prognostic factor in rectal cancer recurrence, and survival is superior [29,30]. Atasoy et al. reported that CRM involvement and the presence of perineural invasion negatively affected the five-year overall survival [20].

Being a single-center retrospective study and a small sample size are the limitations of this study. The small sample size is due to pelvimetry taking too much time and CT not being available. It is necessary to consider all these factors when interpreting the results of this study.

## Conclusions

No benefit was found in predicting CRM involvement from CT pelvimetry in laparoscopic surgery of middle and lower rectal cancer in our study. The predictive factors for CRM involvement were tumor size, tumor height from the anal verge, and gender. In this study, CRM status had no significant effect on survival. Although our study sheds light on this issue, prospective randomized studies are needed.
